# Are unisexual flowers an appropriate model to study plant sex determination?

**DOI:** 10.1093/jxb/eraa207

**Published:** 2020-04-30

**Authors:** Shu-Nong Bai

**Affiliations:** State Key Laboratory of Protein & Plant Gene Research, Quantitative Biology Center, College of Life Sciences, Peking University, Beijing, China

**Keywords:** Heterospory, plant sex, sex differentiation, sexual behaviour, unisexual flowers

**Unisexual flowers are used as a model for studying plant sex determination, but it is a general phenomenon that they result from the developmental inhibition of one type of reproductive organ, which prevents sex cell formation. This should not be categorized as sex differentiation, but rather as the promotion of outcrossing. An alternative view is to regard ‘sex’ as division of types of gametes, which occurs only in eukaryotes, while regarding ‘sex differentiation’ as mechanisms that occur in the soma to ensure heterogametogenesis, which occurs only in multicellular eukaryotes. In the light of these new definitions, distinguishing real- and pseudo-sex differentiation is an interesting issue in plants.**

Since 1998, I have been participating in research using unisexual flowers of cucumber to study plant sex differentiation. Ending in 2016, we published a series of empirical papers reporting the stage at which cucumber stamens or carpels begin to develop abnormally (Bai *et al.*, 2004), what happens in primordial anthers that lead to stamen arrest in female floral buds (Yang *et al.*, 1999; Hao *et al.*, 2003), and how primordial anther-specific damage to DNA is induced organ-specifically during floral bud development (Sun et al., 2010, 2016; Wang *et al.*, 2010; Gu *et al.*, 2011). After more than a decade of investigation, I was convinced that the unisexual flower of cucumber results from the developmental inhibition of one type of reproductive organ, which prevents sex cell formation; therefore, such a phenomenon should not be categorized as sex differentiation, but rather as the promotion of outcrossing, i.e. the promotion of pistil pollination with pollen from other flowers (Bai and Xu, 2012, 2013). This phenomenon is by no means specific to cucumber, but is general among unisexual flowers that have been investigated at the molecular level in maize (Chuck *et al.*, 2007; Acosta *et al.*, 2009; Hartwig *et al.*, 2011; Hayward *et al.*, 2016), persimmon (Akagi *et al.*, 2014), kiwifruit (Akagi *et al.*, 2018, 2019), and asparagus (Harkess *et al.*, 2017).

Ironically, probably because of my publication record, I often received invitations to review manuscripts describing research using unisexual flowers to study plant sex differentiation. I always declined these invitations on the basis that I do not believe that the unisexual flower is the result of sex differentiation in plants, and therefore studies that employ a system of outcross promotion to study the mechanism of sex differentiation are not logically sound. However, since the norm of using unisexual flowers as a model for studying plant sex was not originally proposed by the authors of these papers, it would have been unfair to them if I had rejected their work based on their flawed logic of such a norm. Among the different journals that have asked me to review, only the editors of *JXB* have taken my reply seriously and suggested that I write a viewpoint article to elaborate my opinion to the broader public. I greatly appreciate their consideration, and I would like to take this opportunity to briefly share my thoughts on using a unisexual flower to study sex determination, to touch briefly on the history of doing so, and to revisit the definitions of some relevant terms such as sex, sex differentiation, and sexual behaviors.

## A brief history regarding definition of plant sex

According to Robbins and Pearson (1933) in *Sex in the Plant World*, the first unquestionable experimental demonstration of sex in plants was recorded in 1694 by Rudolf J. Camerarius. Many investigations were subsequently reported and a widely cited reference is that of Yampolsky and Yampolsky (1922). To my knowledge, the earliest scholarly definition of plant sex can be found in Robbins and Pearson’s book, in which they make the following statements, “the vast majority of flowering plants are hermaphrodites. But there are some plants which must, like us, go through life with their lots irretrievably cast, their destinies scaled, as one sex or the other”. In the following paragraph, they write, “what do ‘male’ and ‘female’ really mean? An organism or an organ, or even a trait, is one sex or the other simply because it is associated with the production of the sex cells of that sex”. They go on to conclude, “so in plants a stamen is a male organ because within it are produced the pollen grains, which in turn are the sperm producing plants. And a flower or a plant is male if it bears only stamens. Likewise, a pistil is a female organ, and a flower or a plant is female if it bears only pistils”. Regardless of the lack of a mechanistic interpretation of unisexual flowers, the logic behind the definition of sex was not consistent: whereas they correctly linked the sex organs to the sex cells, i.e. sperm and eggs, they used unisexual flowers to define sex and excluded “hermaphrodite flowers”, even though these bear functional stamens and pistils that both produce sex cells and should therefore also have a sex. It is thus clear that using unisexual flowers to define plant sex is not consistent with defining sex by the association of sex organs with sex cells.

The flaw in the logic of Robbins and Pearson’s statements was recognized by Ainsworth (1999). In his preface to the book he edited entitled *Sex Determination in Plants*, he wrote, “the determination of sex in plants is thus a key developmental decision which leads to the suppression of the program of development of either the male or female organs”, followed by, “unisexual plants offer us the opportunity of investigating plant development in a way which is complementary to the study of flower development in hermaphrodite species”. In his statements, “sex” is no longer associated with sex cells but, conversely, with suppression of male or female organ development. Although he correctly implies that unisexual flowers result from the suppression of either male or female organ development, he avoids clearly defining what sex is in plants.

Despite the different interpretations of the role of unisexual flowers in the context of plant sex outlined in the two points of views considered above, the common problem remains that they both discuss “plant sex” using only flowering plants as examples. However, because non-flowering plants also produce sperm and eggs, how can we discuss “plant sex” without including them?

Fortunately, the literature contains solid descriptions of the plant sex issue. Juarez and Banks (1998) provided a clear and sound definition of sex determination as “the developmental decision that occurs during the plant life cycle that leads to the differentiation of the two organs or cells that produce the two gametes”. Unfortunately, few researchers are aware of this definition, and almost all authors and journals publish papers on unisexual flower development as a mechanism of plant sex determination.

## An alternative view, derived from the concept of sexual reproduction cycle

One thing that might ameliorate our discomfort in acknowledging the incorrect use of unisexual flowers as a model system for deciphering the mechanism of plant sex differentiation is that the interpretation of sex appears to be even less precise in the animal field (Box 1). Regardless of the reasons behind the controversies, from my point of view, these issues can be reconciled if we adopt the concept of the “sexual reproduction cycle” (SRC) that I have proposed (Bai, 2015, 2019). The SRC can be viewed as a specialized “cell cycle” that includes meiosis, heterogametogenesis, and fertilization. The net outcome is a single diploid cell that becomes two cells, which is equivalent to a mitotic cell cycle in terms of the change in cell number. The difference from the mitotic cycle is that the two diploid cells resulting from the SRC contain heritable variation that can aid in the integration of a constantly changing environment. From this perspective, the SRC, or the more widely used term “sexual reproduction”, is essentially a stress-response mechanism (because in unicellular eukaryotes, meiosis and gametogenesis are either individually or both induced by stress; Bai, 2015). This concept revives ideas proposed by Coulter (1914) that sexuality is a method for reproduction under peculiar difficulties that allows more rapid and far more varied evolution, and the proposal by Weismann (1889) that the purpose of sex is to generate genetic variation.

Box 1. **Definitions and descriptions of sex in the literature**

**Table:** 

Type of publication	Year	Definition or description	Reference
Encyclopedia	2013	Sex, the sum of features by which members of species can be divided into two groups—male and female—that complement each other reproductively.	Berrill (2013)
	2017	Organisms of many species are specialized into male and female varieties, each known as a sex.	http://en.wikipedia. org/wiki/Sex
Textbook	2005	Sexual reproduction is the creation of offspring by the fusion of haploid gametes to form a zygote, which is diploid.	Campbell and Reece (2005)
	2010	It should be note that sex and reproduction are two distinct and separable processes. Reproduction involves the creation of new individuals; sex involves the combining of genes from two different individuals into new arrangements.	Gilbert (2010)
Monograph	1982	Sex is a composite process in the course of which genomes are diversified by a type of nuclear division called meiosis, and by a type of nuclear fusion called syngamy, or fertilization.Sex and reproduction are quite distinct processes: sex is a change in the state of cells or individuals, whilst reproduction is a change in their number.	Bell (1982)
	1983	Fisher, 1930: No practical biologist interested in sexual reproduction would be led to work out the detailed consequences experienced by organisms having three or more sexes, yet what else should he do if he wishes to understand why the sexes are, in fact, always two?Sex is defined as gender, male or female. Sex development refers collectively to the various molecular, genetic and physiological processes that produce a male or a female from a zygote of a given genotype and parents in a given environment.	Bull (1983)
	2014	Sex is defined by the occurrence of meiosis.	Beukeboom and Perrin (2014)
Review article	2002	True sex – syngamy, nuclear fusion and meiosis – is found only in eukaryotes.	Cavalier-Smith (2002)
	2013	The core features of sexual reproduction involve: (i) ploidy changes from diploid to haploid to diploid states, (ii) the production of haploid mating partners or gametes from the diploid state via meiosis which recombines the two parental genomes to produce novel genotypes and halves the ploidy and (iii) cell-cell recognition between the mating partners or gametes followed by cell-cell fusion to generate the diploid zygote and complete the cycle.	Heitman *et al.* (2013)

If the SRC is accepted to be an essential and conserved process in all eukaryotes and ultimately a mechanism for adapting to unpredictable environmental stresses, it becomes easy to clarify terms such as sex, sex differentiation, and sexual behavior as follows. “Sex”, in line with its Latin root *sexus*, meaning divide, refers to heterogametogenesis (or heterogametes), a phenomenon that exists in unicellular and multicellular eukaryotes. “Sex differentiation”, which is present in multicellular eukaryotes, refers to mechanisms that occur in soma to ensure heterogametogenesis. And “sexual behavior”, also evolved by multicellular eukaryotes, refers to the various mechanisms within the soma that ensure that gametes meet and that enhance the selection of adaptive traits (Bai, 2019). From this perspective, unisexual flowers should not be considered to represent a mechanism of sex differentiation, because the known mechanisms of unisexual flower production all involve preventing, not ensuring, gametogenesis by interrupting either stamen or carpel development (Chuck *et al.*, 2007; Acosta *et al.*, 2009; Hartwig *et al.*, 2011; Akagi et al., 2014, 2018, 2019; Hayward *et al.*, 2016; Harkess *et al.*, 2017). The unisexual flowers that have been investigated at the molecular level to date should rather be considered to represent mechanisms of outcross promotion (Bai and Xu, 2012, 2013).

To what then should sex differentiation in plants phenotypically refer? Based on the above definition of sex differentiation, the differentiation of the archegonium and antheridium in bryophytes and pteridophytes should be categorized as sex differentiation. According to the hypothesis concerning the origin of heterospory proposed by Wang and Bai (2019), stamen and ovule differentiation in spermatophytes should be considered as pseudo-sex differentiation. The rationale for this proposal is that stamens and ovules originate from heterosporangia, which do not directly lead to heterogametogenesis, but rather to meiosis. However, given the severe reduction of the gametophytic phase in spermatophytes, with no differentiation of the archegonium and antheridium, the function of heterogametogenesis is canalized into post-heterospore differentiation.

The use of unisexual flowers as a model for studying plant sex differentiation has been pursued for at least 87 years, since Robbins and Pearson published their book in 1933. However, sex and sex differentiation have existed in nature for billions and hundreds of millions of years, respectively. It is forgivable to interpret phenomena incorrectly due to lack of information, but it is unforgivable to insist on an incorrect interpretation that no longer reflects the experimental data in a logically consistent manner.
